# Metal (II) Complexes of Fluconazole: Thermal, XRD and Cytotoxicity Studies

**DOI:** 10.22037/ijpr.2020.1101142

**Published:** 2020

**Authors:** Syed Imran Ali, Zi-Ning Lei, Mohsin Ali, Konatsu Kojima, Mansoor Ahmed, Richard Peng, Dong-Hua Yang, Syed Moazzam Haider, Seyed Abdulmajid Ayatollahi, Zhe-Sheng Chen

**Affiliations:** a *Department of Applied Chemistry and Chemical Technology, Faculty of Science, University of Karachi, Karachi-75270, Pakistan. *; b *Department of Pharmaceutical Sciences, College of Pharmacy and Health Sciences, St. John’s University, Queens, NY 11439, USA. *; c *Department of Chemistry, Faculty of Science, University of Karachi, Karachi-75270, Pakistan.*; d ^*d*^ *Department of Pharmaceutical Chemistry, Faculty of Pharmacy, University of Karachi, Karachi-75270, Pakistan. *; e *Industrial Analytical Center (ICCBS), University of Karachi, Karachi-75270, Pakistan.*; f ^*f*^ *Phytochemistry Research Center, Shahid Beheshti Univercity of Medical Sciences, Tehran, Iran. *; g *Department of Pharmacognosy, School of Pharmacy, Shahid Beheshti University of Medical Sciences, Tehran, Iran.*

**Keywords:** Fluconazole, Metal complexes, Anticancer activity, X-ray diffraction, Thermogravimetry (TGA), Differential scanning calorimetry (DSC)

## Abstract

We report thermal, X-ray diffraction (XRD) and cytotoxicity studies of complexes of fluconazole (FCZ) with Cu (II), Fe(II), Cd(II), Co(II), Ni(II), and Mn(II). From XRD measurements, FCZ and its metal complexes were identified as polycrystalline. Marked differences in the X-ray patterns of drug and its metal complexes revealed that the complexes are indeed different compounds and not just the mixture of the starting materials. Unlike pristine FCZ, which did not exhibit cytotoxicity, three complexes derived from Fe(II), Cu(II) and Co (II) proved to be effective in the cytotoxicity assay. The Cu(II)-FCZ exhibited significant activity against SNB-19, HCT-15, COLO-205, and KB-3-1 cell lines, while Fe(II)-FCZ and Co(II)-FCZ were found cytotoxic only to KB-3-1 cell line. For the pure FCZ, thermogravimetry revealed massive weight loss in the temperature range of 215 to 297 °C, due to the volatilization of FCZ. All the complexes followed multi-stage degradation profiles, eventually resulting in the formation of metal oxides. For pure FCZ, differential scanning calorimetry revealed melting point at 137 °C, followed by two further endothermic transitions at 294 °C and 498.44 °C representing the volatilization and subsequent degradation of FCZ, respectively. The absence of endothermic FCZ melting peak at around 137 °C indicates that the complexes represent different compounds. All complexes exhibit endothermic transitions at around 240-300 °C, representing melting and removal of ligand moiety, followed by another endothermic transition at around 498-499 °C, representing the ligand decomposition.

## Introduction

Over the last few decades, growing resistance of microorganisms against existing drugs have been a significant concern for researchers as it poses a considerable burden on the health departments of nations around the world (1-3). Substantial increase has been observed in the population of patients suffering from fungal infections, particularly infecting immune compromised patients, such as those infected with HIV/AIDS, or with a neoplastic condition, transplant recipients, patients under treatment in intensive care units and those which are undergoing chemotherapies (4-13). The growing trend in the population of patients at risk posed a serious challenge to the medical community and triggered the endeavors to explore new strategies to improve efficiency and efficacy of existing antifungals (5, 6). 

Among the antifungals introduced in clinical practice, fluconazole (FCZ) remains the most widely explored owing to many advantages it offers (6, 14). Due to its excellent antifungal activity coupled with low toxicity, excellent pharmacokinetics and bioavailability, FCZ was considered to be the standard treatment against various fungal infections for many years since it became available for clinicians in 1990s (6, 15-18). The hydro-soluble nature renders it suitable for intravenous admiration (6). Besides, it demonstrated excellent gastrointestinal absorption and spread easily by diffusion throughout the entire body, including cerebrospinal fluid (19, 20). For treating fungal infections in human, FCZ has demonstrated significantly better clinical and mycological cure rate compared to itraconazole in oropharyngeal candidiasis (21). Oral suspensions of FCZ are widely used in oral pseudomembranous candidiasis because of its good adhesion to the surface of the oral mucosa and a rapid symptomatic response (22-26). Its efficacy has also been demonstrated in immune compromised patients, such as those which are HIV-infected, or with a neoplastic condition (21, 27 and 28). FCZ also exhibits the activity against *Candida lusitaniae. *For patients suffering from candidial osteomyelitis who are unable to start or complete the required course of amphotericin B, FCZ is a reasonable alternative and has been successful in achieving desired treatment results (29, 30).

Despite the set of favorable properties it offers, FCZ is still not considered as the perfect antifungal and has certain drawbacks which needs to be overcome. For instance, just like the other members of Azole family of antifungals, it has some non-negligible interactions with certain drugs which can cause a decrease in drug concentration or, to some extent, an increased toxicity (31). Besides, its ineffectiveness against emerging pathogens such as *Scedosporium*, *Fusarium*, and *Mucorales* has also been reported (32). Since its emergence in the market, FCZ simulated extensive use against various fungal infections and chemoprophylaxis (33-35). Afterwards, over-prescription by physicians led to an increase in resistance to FCZ in a high percentage of patients (15, 36-39). Well established clinical and commercial importance highlights the need to explore new strategies and formulations to improve efficiency and efficacy of FCZ.

During the past decade, synergic effect of metal ions on the activity and efficacy of various drugs has been reported in several research papers. Drug-metal ion complexes have proven their efficacy in various fields of health such as anticancer chemotherapeutic agents, antacids, and anti-rheumatics (40, 41). With the aim to improve efficiency and efficacy of FCZ, we recently started research work to explore the synthesis of metal complexes of FCZ. A series of complexes of fluconazole with Cu (II), Fe(II), Cd(II), Co(II), Ni(II) and Mn(II) were synthesized and the morphological, spectroscopic, and antifungal properties were thoroughly reported (40). In this contribution, we further extend our previous study on these complexes to explore their thermal, X-ray diffraction, and cytotoxicity properties.

## Experimental

*Materials*

The fluconazole (FCZ) sample was kindly provided by NabiQasim Pharma (PVT) Ltd, Karachi, Pakistan. Hydrated chlorides of copper, iron, manganese, cadmium, cobalt, and nickel (Merck, Germany) were all used as received. Methanol (analytical grade, Merck, Germany) was used as received. For all experiments, freshly distilled water was used.

*Synthesis of the Complexes*

**Scheme 1:**
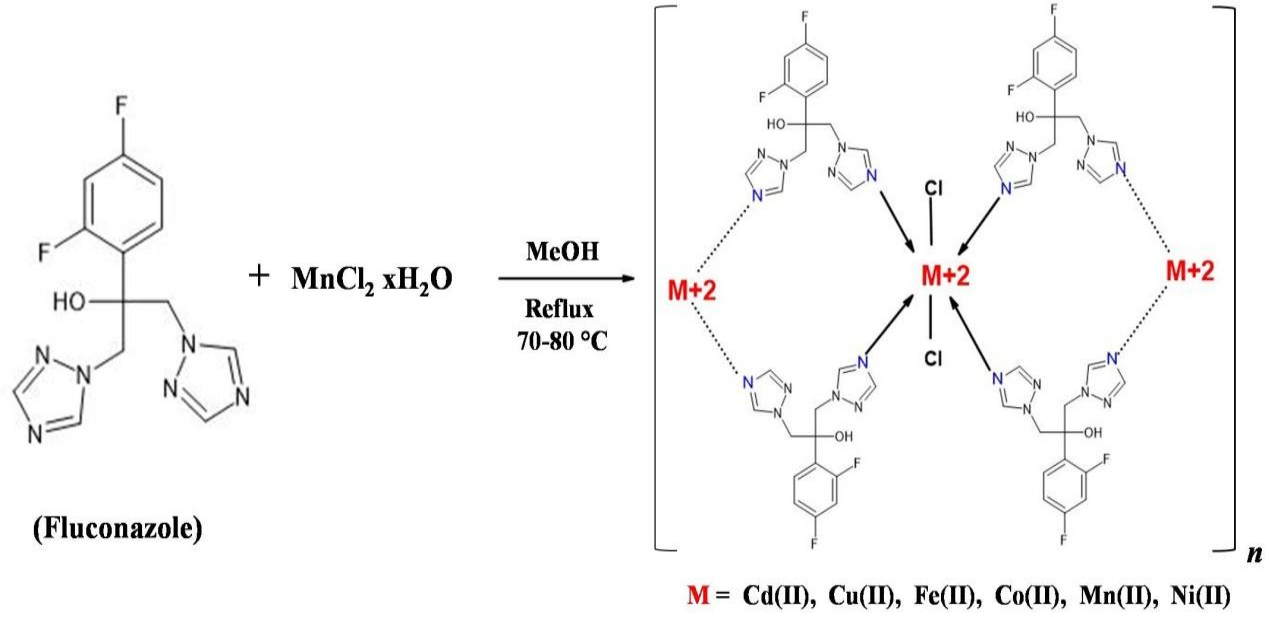
Synthesis of metal complexes of fluconazole (FCZ)

A series of metal complexes of FCZ with Co, Mn, Fe, Ni, Cu, and Cd were synthesized. Details of the synthesis procedure and characterizations of the resulting complexes have already been described in our previous paper (40). A general synthesis scheme is depicted in Scheme 1.

*Characterization*

X-ray diffraction studies were performed to explore the crystallinity of fluconazole and its metal complexes using a Bruker AXS D8 (Germany) X-Ray diffractometer operated at 40 kV and 60 mA and equipped with a Cu-Kα1 radiation (1.54 Å). The data were recorded at a scanning speed of 5◦/min in the range of 10° < 2θ < 60° using a step size of 0.02°/point. All samples were analyzed as dry powder.

Thermal properties of complexes were studied using differential scanning calorimetry (DSC) performed on a TA Instruments STD Q600 system under nitrogen atmosphere (50 kPa pressure). For all measurements, around 10 to 12 mg of powder samples, enclosed in DSC pans, were heated from room temperature to 700 °C at a scan rate of 10 °C min^-1^. The parameters, such as onset temperature and peak temperature, were recorded from heating scans. Thermogravimetric analysis (TGA) was performed on a TA Instruments STD Q600 system using approximately 10 to 12 mg samples placed in crucibles. The samples were heated from room temperature to 700 °C at a heating rate of 10 °C min^-1^ in nitrogen atmosphere.

Four cell lines including human astrocytoma SNB-19, human Dukes’ type C colorectal adenocarcinoma HCT-15, human Dukes’ type D colorectal adenocarcinoma COLO-205, and human epidermoid carcinoma KB-3-1 were obtained from the American Type Culture Collection (ATCC, Manassas, VA). The cell lines were grown in DMEM supplemented with 10% FBS and 1% penicillin/streptomycin in a humidified incubator at 37 °C with 5% CO_2_.

MTT (3-[4,5-dimethylthiazol-2-yl]-2,5-diphenyltetrazolium bromide) assay was used to evaluate the cytotoxicity of the test compounds. The cells were inoculated in 96-well plates at the density of 5,000 cells per well and allowed to adhere and grow for 24 h. The test compounds in different concentration were incubated with the cells for 72 h. After drug incubation, 4 mg/mL MTT solution was added to the cells by 20 μL per well followed by a four-hour incubation. The cell viability was measured by reduction of the yellow dye MTT to a blue formazan product that were dissolved in DMSO (42). The absorbance of the blue dissolved formazan crystals in the viable cells was measured at 570 nm by using accuSkan™ GO UV/Vis Microplate Spectrophotometer (Fisher Sci., Fair Lawn, NJ). The IC_50_ values (50% inhibitory concentration) were calculated.

## Results and Discussion

*X-Ray Diffraction*

**Figure 1:**
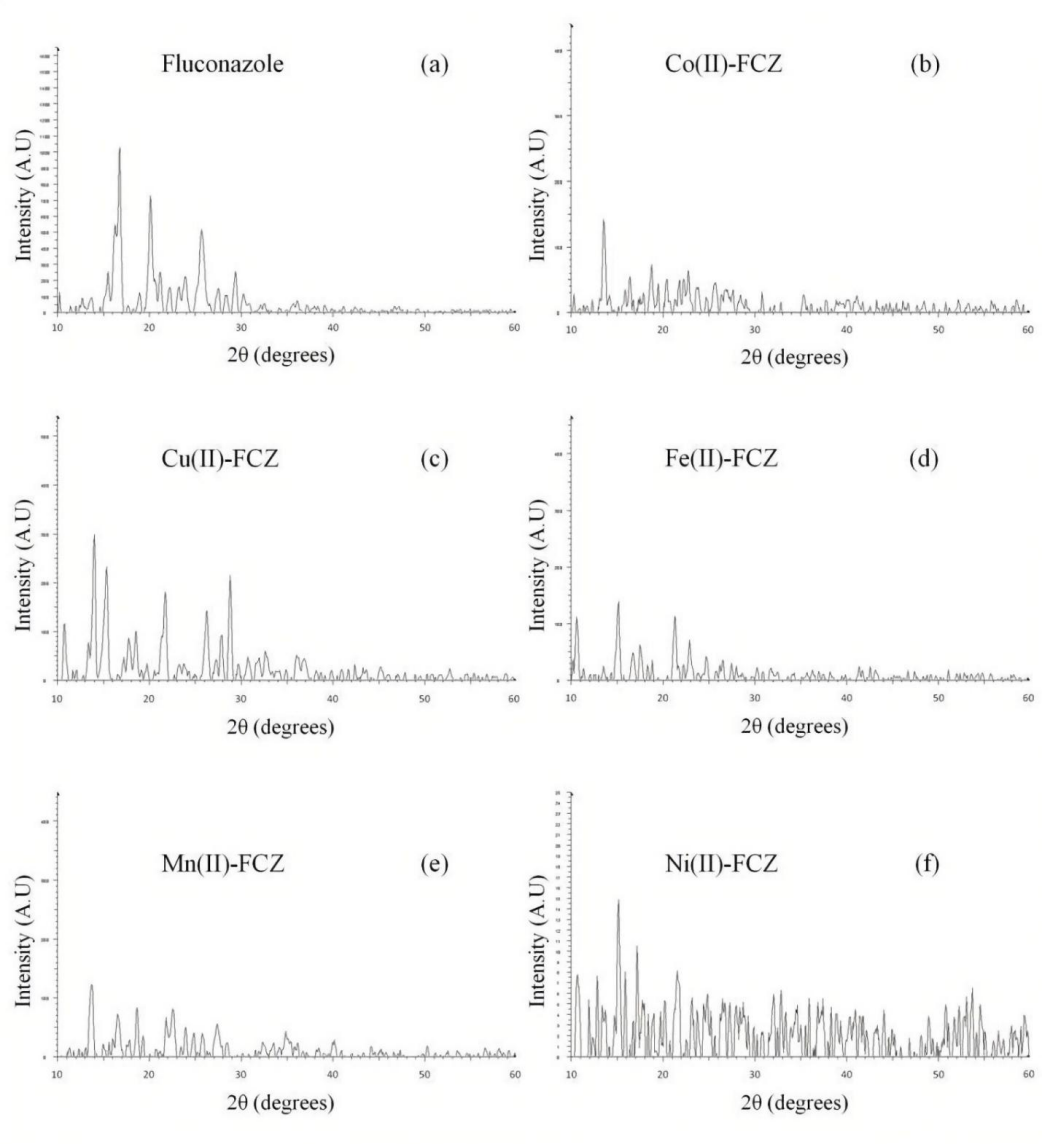
**X-ray diffractograms of (a) pristine FCZ, (b)** **Co (II)-FCZ** **, (c)** **Cu(II)-FCZ** **, (d)** **Fe (II)-FCZ** **, (e)** **Mn (II)-FCZ** **and (f)** **Ni (II)-FCZ** **complexes**

To explore the structure of the FCZ and its metal complexes, X-ray diffraction technique was used. The resulting X-ray patterns of pure FCZ and its metal complexes are shown in Figure 1. For the pure FCZ sample (Figure 1a), prominent diffraction peaks in the range of 2θ = 10–60ᵒ are evident clearly indicating a polycrystalline nature. It has been reported earlier that FCZ existed in at least two polymorphic forms which exhibit granular and flake-like slabs morphologies. The observed pattern agrees fairly well with that reported by Satish *et al.*, although there are few more spikes, which are likely due to the presence of more than one polymorphic form (42, 43). Consequently, the raw FCZ was identified as a mixture of polymorphs. 

The diffractograms obtained for the metal complexes Co(II)-FCZ, Cu(II)-FCZ, Fe(II)-FCZ, Mn(II)-FCZ, and Ni(II)-FCZ are depicted in Figures 1b-1f, respectively. By comparing the obtained X-ray powder diffraction patterns given in Figure 1, it can be easily seen that the pattern obtained for the pure FCZ sample (Figure 1a) differs drastically from those obtained for all its metal complexes. Thus, it can be inferred that each complex represents a definite compound of a definite structure and not merely the mixture of the starting materials (44). Besides, all complexes exhibited diffraction peaks at various angles with a lower intensity compared to the pure drug showing their crystalline nature with smaller particle sizes.

*Thermal Stability*

**Figure 2:**
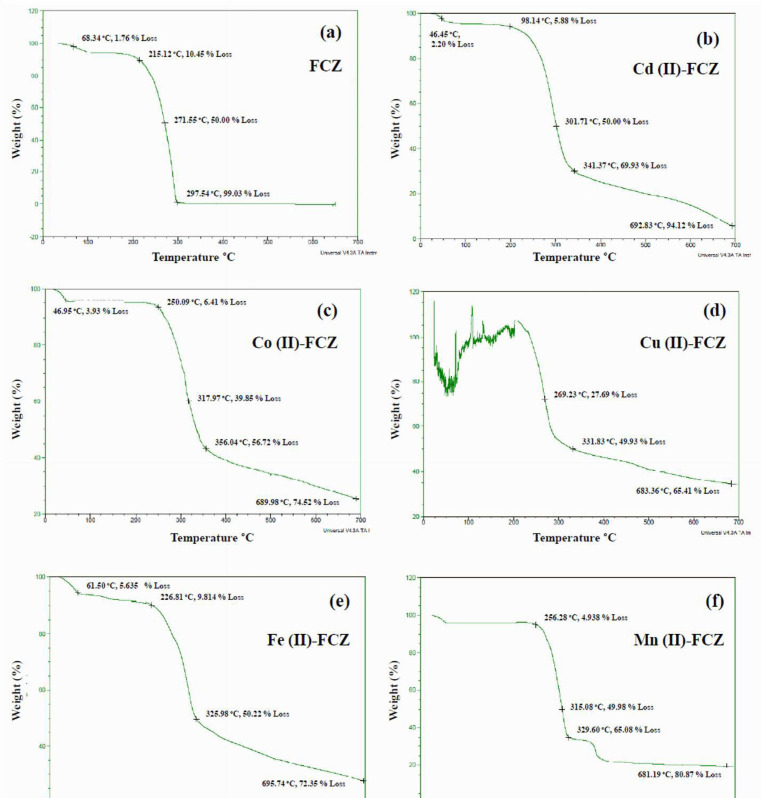
**Thermo gravimetric profiles of (a) FCZ, (b)** **Cd (II)-FCZ** **, (c)** **Co (II)-FCZ** **, (d)** **Cu (II)-FCZ** **, (e)** **Fe (II)-FCZ** **, (f)** **Mn (II)-FCZ, and** **(g)** **Ni (II)-FCZ** **complexes**

Thermal stabilities of FCZ and its metal complexes were explored using thermogravimetric analysis (TGA) which was performed on powder samples under inert atmosphere employing a heating rate of 10 °C min^-1^. The resulting thermograms are shown in Figure 2. For the pristine drug sample, a weight loss of around 1.8% was observed before the melting temperature in the range of 65–110 °C, which corresponds to the loss of water content. Considering the molecular weights of water and FCZ, and the percent weight loss, it can be inferred that the raw FCZ employed in this study is likely a mixture of different polymorphs and not solely consist of a monohydrate (45, 46). This result is consistent with the XRD findings which also revealed the presence of more than one polymorphic form. 

Upon further heating, FCZ undergoes a melting transition at around 135 to 138 °C. The drug then remained stable up to 215°C until a massive weight of around 99% was observed in the temperature range of 215 to 297 °C. The observed massive weight loss, as previously reported by Moura *et al.* is attributed to the volatilization of molecular FCZ (47). The thermogravimetric analysis were also performed for Cd (II)-FCZ, Co (II)-FCZ, Cu(II)-FCZ, Fe (II)-FCZ, Mn(II)-FCZ, and Ni (II)-FCZ complexes and the results are presented in Figures 2b-2g. As was expected, the decomposition of all the complexes eventually resulted in the formation of metal oxide which demonstrates stability throughout the temperature range explored. All complexes exhibit multi stage degradation profiles which started with the initial loss of water molecules followed by losses of ligand molecules. Remarkably, compared to pure FCZ, the complexes exhibit better thermal stability and resulted in a substantial residual mass even after heating to 700 °C.

*Differential Scanning Calorimetry (DSC)*

**Figure 3:**
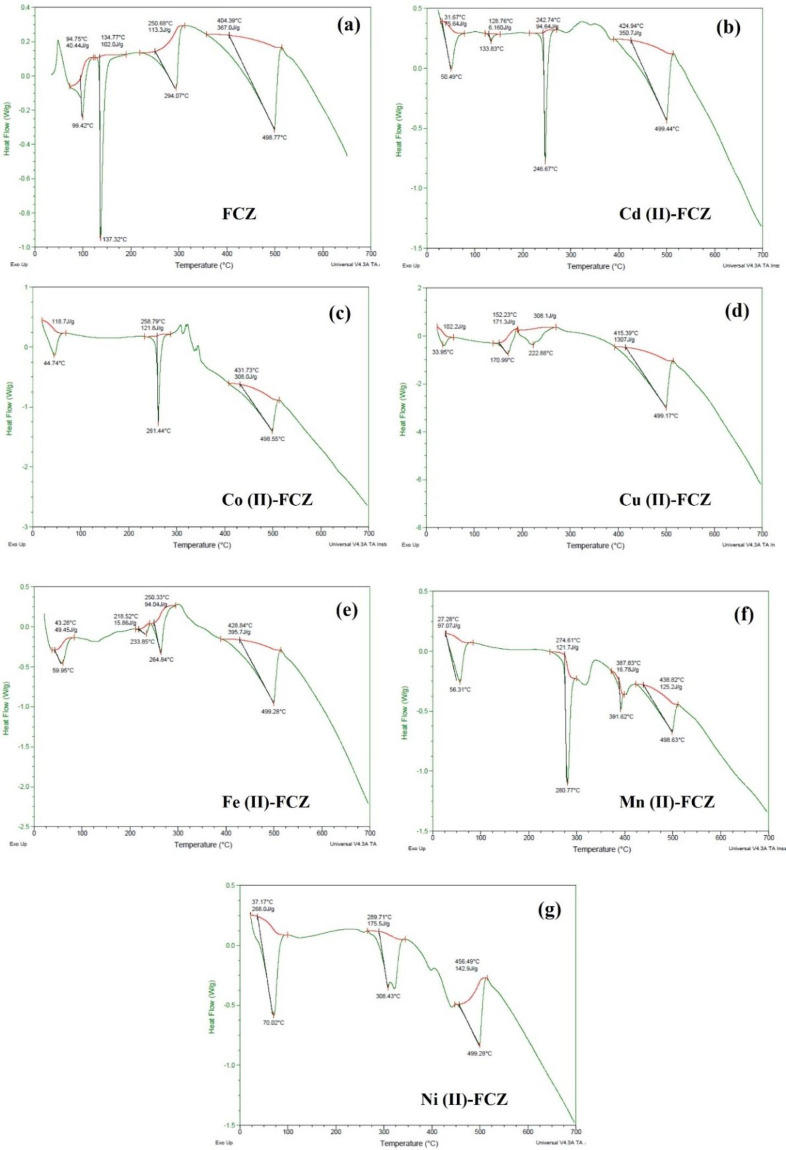
**DSC curves for (a)** **FCZ, (b)** **Cd (II)-FCZ** **, (c)** **Co (II)-FCZ** **, (d)** **Cu (II)-FCZ** **, (e)** **Fe (II)-FCZ** **, (f)** **Mn (II)-FCZ and** **(g)** **Ni (II)-FCZ** **complexes**

The DSC patterns recorded for FCZ and its complexes are shown in Figure 3. An endothermic peak located at around 100 °C in the calorimetric curve of FCZ (Figure 3a) corresponds to the dehydration process. As the melting points of the three known polymorphic forms of FCZ have been reported to fall in the range of 135 to 140 °C, the endothermic transition detected at 137 °C is clearly representing the melting transition (47, 48). Endothermic transition observed beyond melting likely corresponds to the volatilization of molecular FCZ at 294 °C and its subsequent degradation at 498.44 °C (47). The calorimetric curves were also recorded for all the six complexes (Cd (II)-FCZ, Co (II)-FCZ, Cu (II)-FCZ, Fe (II)-FCZ, Mn (II)-FCZ, and Ni (II)-FCZ). For all the complexes explored, the observed endothermic transitions in the temperature range of 30 to 70 °C correspond to the loss of water molecules from the crystals (49, 50). A striking feature of the calorimetric curves of the complexes is the absence of endothermic melting peak of pure FCZ which indicates that these complexes represent definite compounds and are not merely the mixture of the starting materials. Further, endothermic peaks representing the melting and subsequent removal of ligand moiety occurred for Cd (II)-FCZ at 246.67 °C, Co (II)-FCZ at 261.44 °C, Cu (II)-FCZ at 170 °C and 222.88 °C, Fe (II)-FCZ at 264.34 °C, Mn (II)-FCZ at 290.77 °C and Ni (II)-FCZ at 308.43 °C. In case of all the complexes, endothermic transitions occurred at around 498-499 °C corresponding to the decomposition of ligand after which the complexes exhibit gradual decomposition up to 700 °C.

*Anticancer Activity*

**Figure 4:**
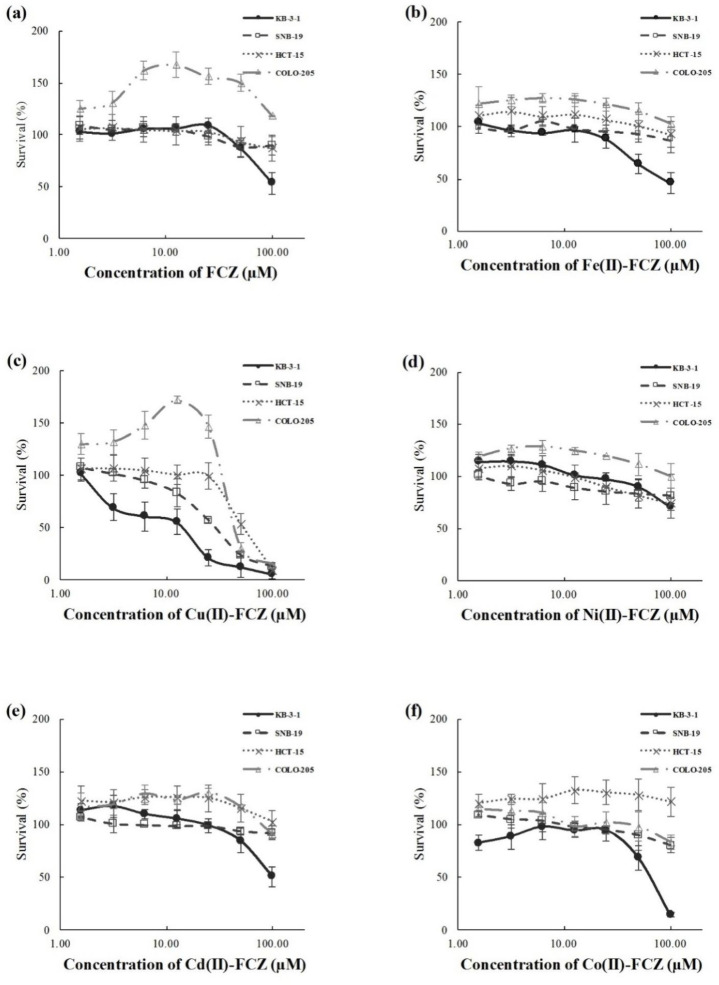
**The cytotoxic effects of (a) FCZ, (b) Fe(II)-FCZ, (c), Cu(II)-FCZ, (d) Ni(II)-FCZ, (e) Cd(II)-FCZ, and (f) Co(II)-FCZ on the KB-3-1, SNB-19, HCT-15 and COLO-205 cell lines as determined by MTT assays. A serial concentrations used in various comp**

The IC_50_ values of the FCZ and its metal complexes on the human cancer cells used were summarized in Table 1. For the two types of human colorectal adenocarcinoma cells HCT-15 and COLO-205 cells, only Cu(II)-FCZ had slight cytotoxic effects, with similar IC_50 _values (mean ± standard deviation) of 60.10 ±7.85 μM, and 60.90 ± 4.58 µM, respectively. Also, only Cu(II)-FCZ had mild cytotoxicity on SNB-19 cells, but with a relatively lower IC_50_ value of 27.80 ± 4.16 µM. KB-3-1 cells exhibited higher sensitivity to the drugs than the other three cell lines. Among the seven compounds, Fe(II)-FCZ, Cu(II)-FCZ, and Co(II)-FCZ had IC_50 _values lower than 100 µM on KB-3-1 cell line, which were 81.33 ± 11.35 µM, 13.04 ± 5.72 µM, and 62.03 ± 19.84 µM, respectively (Figure 4).

The results reflected that metal cations, in the form of drug complexes with organic ligand, act as a critical role in anticancer activity. Previous study showed that the complexes are able to stabilize the cleavable complex formed between enzyme and DNA, meanwhile control the replication and transcription of DNA in malignant tumour cells (44). Therefore, a complex with cation metal would show more active anticancer efficiency than the ligand alone. In this study, we also observed enhanced cytotoxic effects of metal complex than the parent compound FCZ on cancer cell lines. The mechanism may be related to the charge of metal and the high reactivity of the complex due to unpaired electrons, which may lead to superoxide dismutase (SOD) mimic activity and DNA cleavage activity that further results in cell apoptosis (51). This has been proved by the previous study with other azole compounds and metal complexes. 

For example, a recent research showed that benzotriazole based Fe(III)-salen-like complex displayed remarkable anticancer activity against human chronic myelogenouserythroleukemia cell line and breast adenocarcinoma cell line, and further mechanistic studies supported that the resulting cancer cell apoptosis was probably led by certain superoxide dismutase (SOD) mimic activity and the subsequent local imbalance in superoxide/hydrogen peroxide levels(48). However, in our observation, not all metal complexes had significant anticancer effects, and a complex may not show cytotoxicity on all cancer cell lines, indicating that different metals may have different mechanisms of effects, which requires more researches in the future to uncover other findings.

**Table 1:** Cytotoxicity of FCZ and metal complexes of FCZ on four human cancer cell lines

**Drug**	**IC**_50_ ** ± SD (μM)**			
	**SNB-19**	**HCT-15**	**COLO-205**	**KB-3-1**
FCZ	>100	>100	>100	>100
Fe(II)-FCZ	>100	>100	>100	81.33 ± 11.35
Cu(II)-FCZ	27.80 ± 4.16	60.10 ± 7.85	60.90 ± 4.58	13.04 ± 5.72
Ni(II)-FCZ	>100	>100	>100	>100
Mn(II)-FCZ	>100	>100	>100	>100
Cd(II)-FCZ	>100	>100	>100	>100
Co(II)-FCZ	>100	>100	>100	62.03 ± 19.84

## Conclusion

Clinical and commercial importance of fluconazole (FCZ) has become an inspiration for researchers to explore new strategies and formulations to improve its growing ineffectiveness as antifungal. Complexation of drugs with metal ions is a well-established approach in medicinal chemistry to improve the efficacy of various drugs. The present contribution was intended to explore six complexes of FCZ with Cu (II), Fe(II), Cd(II), Co(II), Ni(II), and Mn(II) for their thermal, XRD, and cytotoxicity properties.

Our results revealed that pure FCZ and its metal complexes were of polycrystalline nature. Contrary to the pure FCZ, three complexes demonstrated cytotoxicity against four human cancer cell lines used in the cytotoxicity assay. The Cu (II)-FCZ complex had cytotoxic activity against all four cancer cells, while Fe (II) and Co (II) complexes of FCZ showed some cytotoxic activity against KB-3-1 cancer cell, which implied the important role of metal complexes in anticancer activity. In case of pure FCZ, thermogravimetry revealed massive weight loss in the temperature range of 215 to 297 °C, due to the FCZ volatilization. The complexes; however followed multi stage degradation profiles, eventually resulting in the formation of metal oxides. From differential scanning calorimetry, the melting transition for pure FCZ was identified at 137 °C. Remarkably, this transition was not observed for all the six complexes indicating that these complexes represent definite compounds. Additional endothermic transitions for pure FCZ and its metal complexes were also observed.

Although not all metal complexes showed effectiveness, the obtained results indicate activity of Cu (II)-FCZ, Fe (II)-FCZ, and Co (II)-FCZ for the chosen strains. It can be said that the FCZ metal complexes embody a starting point towards the development and optimization of more effective anticancer and antifungal drugs and will likely lead to new direction for studies of novel antifungal formulations. More studies on evaluation of these metal complexes and further preparation of different derivatives are needed. 
